# Protein-Pacing Caloric-Restriction Enhances Body Composition Similarly in Obese Men and Women during Weight Loss and Sustains Efficacy during Long-Term Weight Maintenance

**DOI:** 10.3390/nu8080476

**Published:** 2016-07-30

**Authors:** Paul J. Arciero, Rohan Edmonds, Feng He, Emery Ward, Eric Gumpricht, Alex Mohr, Michael J. Ormsbee, Arne Astrup

**Affiliations:** 1 Human Nutrition and Metabolism Laboratory, Health and Exercise Sciences Department, Skidmore College, Saratoga Springs, NY 12866, USA; redmonds@skidmore.edu (R.E.); fhe@csuchico.edu (F.H.); emeryward@gmail.com (E.W.); 2 Department of Kinesiology, California State University-Chico, Chico, CA 95929, USA; 3 Isagenix International LLC, Gilbert, AZ 85297, USA; eric.gumpricht@isagenixcorp.com (E.G.); alex.mohr@isagenixcorp.com (A.M.); 4 Department of Nutrition, Food and Exercise Sciences, Florida State University, Tallahassee, FL 32306, USA; mormsbee@fsu.edu; 5 Department of Biokinetics, Exercise and Leisure Sciences, University of KwaZulu-Natal, Durban 4000, South Africa; 6 Department of Nutrition, Exercise and Sports, University of Copenhagen, København 1017, Denmark; ast@nexs.ku.dk

**Keywords:** protein-pacing, caloric restriction, intermittent fasting, abdominal obesity, heart healthy

## Abstract

Short-Term protein-pacing (P; ~6 meals/day, >30% protein/day) and caloric restriction (CR, ~25% energy deficit) improves total (TBF), abdominal (ABF) and visceral (VAT) fat loss, energy expenditure, and biomarkers compared to heart healthy (HH) recommendations (3 meals/day, 15% protein/day) in obese adults. Less is known whether obese men and women respond similarly to P-CR during weight loss (WL) and whether a modified P-CR (mP-CR) is more efficacious than a HH diet during long-term (52 week) weight maintenance (WM). The purposes of this study were to evaluate the efficacy of: (1) P-CR on TBF, ABF, resting metabolic rate (RMR), and biomarkers between obese men and women during WL (weeks 0–12); and (2) mP-CR compared to a HH diet during WM (weeks 13–64). During WL, men (*n* = 21) and women (*n* = 19) were assessed for TBF, ABF, VAT, RMR, and biomarkers at weeks 0 (pre) and 12 (post). Men and women had similar reductions (*p* < 0.01) in weight (10%), TBF (19%), ABF (25%), VAT (33%), glucose (7%–12%), insulin (40%), leptin (>50%) and increase in % lean body mass (9%). RMR (kcals/kg bodyweight) was unchanged and respiratory quotient decreased 9%. Twenty-four subjects (mP-CR, *n* = 10; HH, *n* = 14) completed WM. mP-CR regained significantly less body weight (6%), TBF (12%), and ABF (17%) compared to HH (*p* < 0.05). Our results demonstrate P-CR enhances weight loss, body composition and biomarkers, and maintains these changes for 52-weeks compared to a traditional HH diet.

## 1. Introduction

Dietary interventions remain the cornerstone of treating obesity. However, one systematic review of weight loss interventions (including diet only, diet combined with exercise, meal replacements, very-low calorie diets, and other weight-loss medications) revealed that weight loss tended to plateau after six months, with mean weight losses corresponding to 5%–9% initial body weight [1]. Not surprisingly, the failure of established dieting strategies for weight loss, and more importantly weight loss maintenance, has prompted an increase in lower-calorie nutritional interventions to facilitate weight loss and reduce weight regain [2,3,4,5,6], but without much success [7]. 

Additional therapeutic avenues for enhancing weight loss and body composition have been demonstrated by nutritional interventions such as protein-pacing (P, 4–6 meals/day, >30% protein/meal), low-carbohydrate (<175 grams/day), and heart healthy (HH) dietary regimens, which have also been associated with improvements in adipokines, blood lipids, insulin and other cardiovascular biomarkers [8,9,10,11,12]. A protein-pacing diet incorporates timed-daily ingestion of protein-rich meals from supplementation and/or whole food sources providing approximately 0.3 g/kg body weight per meal or >1.4 g/kg body weight per day [8]. One explanation for the potential benefit of a P diet for supporting weight loss and weight maintenance may be via augmentation of energy expenditure and/or prevention of resting metabolic rate (RMR) reductions that typically accompany weight loss [8,13]. Additional promising strategies involving caloric restriction (CR, >25% energy deficit), which encompass a variety of regimens that manipulate total caloric intake and timing of food consumption, include intermittent fasting (IF, typically 1–2 days/week) to promote weight loss and improve body composition. Indeed, several clinical studies have showed CR approaches, utilizing IF, to be effective in reducing body weight (BW), total (TBF) and abdominal (ABF) fat mass (including visceral adipose tissue, VAT) while positively influencing inflammatory and adipokine biomarkers in overweight/obese subjects [14,15,16]. A longer study lasting 12 to 24 weeks showed IF to reduce body weight (3%–9%) and body fat, and favorably alter cardiometabolic parameters [17]. 

Despite the demonstrated benefits of short and intermediate P and CR (including IF) interventions, there is little research comparing the effects of a combined P-CR on BW, TBF, ABF, RMR and adipokines between obese men and women during short-term (12 weeks) weight loss (WL). For example, studies suggest men lose more abdominal fat mass than women during WL [18,19], although this is not universal [8,20]. Moreover, it is unknown whether a P-CR dietary approach is more successful than a traditional HH higher carbohydrate intervention during long-term (one year) weight maintenance (WM). 

Accordingly, the first aim of this study was to examine sex-specific responses to short-term (weeks 0–12) WL, including body composition, resting metabolic rate (RMR), and plasma biomarkers following a P-CR diet intervention. The second aim was to extend beyond the WL phase and include a long-term (weeks 13–64) WM phase to compare a modified P-CR (mP-CR) to a HH dietary intervention. We hypothesized, based on our previous findings in obese men and women [8], that P-CR would improve body composition and favorably alter metabolism and biomarkers similarly in obese men and women following short-term WL. Additionally, we hypothesized that these favorable improvements would be better sustained following a long-term mP-CR than a HH diet in obese men and women. 

## 2. Materials and Methods

### 2.1. Participants

This study enrolled 128 individuals from the Saratoga Springs, NY area. Potential participants responded to flyers, local newspapers, or emails advertising the study. The number of subjects initially screened was 108, of which 43 were eligible for our study. Participants were healthy, nonsmoking, obese/overweight men and women. A comprehensive medical examination/history assessment was performed by their physicians to rule out any previous cardiovascular or metabolic disease. For at least six months prior to the start of the study, all subjects were either sedentary or lightly active (<30 min, 2 days/week of organized physical activity), overweight or obese (BMI > 27.5 kg/m^2^; % body fat > 30%), weight stable (± 2 kg), and middle aged (30–65 years). Every participant provided informed written consent in accordance with the Skidmore College Human Subjects review board prior to participation. The study was approved by the Human Subjects Institutional Review Board of Skidmore College (IRB#: 1307-347). All experimental procedures were performed in adherence with related New York State regulations and the Federal Wide Assurance, which are consistent with the National Commission for the Protection of Human Subjects of Biomedical and Behavioral Research, and in agreement with the Helsinki Declaration (revised in 1983). This trial was registered at clinicaltrials.gov as NCT02525419. 

### 2.2. Experimental Design

#### Study Timeline

**Figure 1:**
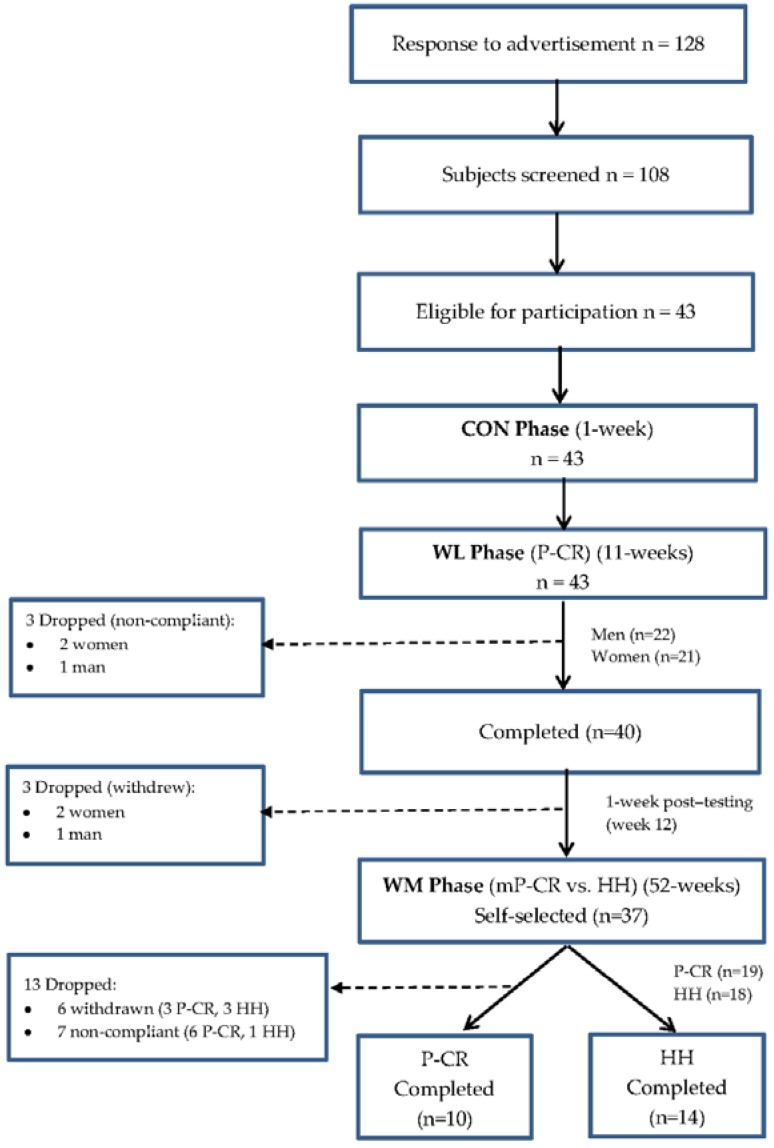
CONSORT flow diagram for study: Weight Loss (WL)-Phase 1 and Weight Maintenance (WM)-Phase 2. CON = control period.

Subjects were registered as a single cohort in this 64 week dietary regimen, splitting into two consecutive intervention phases: (a) 12-week WL P-CR diet (weeks 0–12; 1-week baseline control, 10-week WL, 1-week post testing); and (b) a 52-week WM phase (weeks 13–64) comparing a modified P-CR (mP-CR) with a HH dietary plan (Figure 1 shows the CONSORT study flow chart).

**Figure 2:**
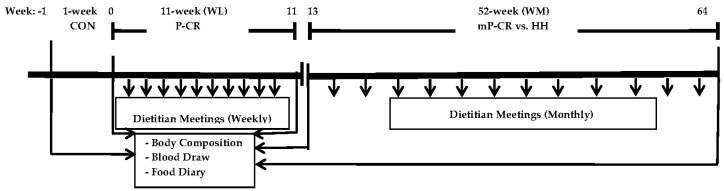
Study Timeline for Weight Loss-Phase 1 and Weight Maintenance-Phase 2.

All laboratory testing procedures (see below) were performed at baseline control (week 0), week 12, and week 64 (Figure 2 shows the study timeline and assessment periods).

During the 1-week baseline control, subjects maintained a stable body weight by consuming a similar caloric intake as their pre-enrollment caloric intake, while maintaining their sedentary lifestyle. Following control baseline testing, participants were provided with detailed instructions on their WL dietary guidelines and scheduled weekly meetings with a dietitian. The WL phase began with all participants following a 10 week controlled P-CR intervention as detailed below. Prior to the completion of the WL phase, researchers decided to extend the study an additional 52 weeks with full IRB approval and consent from all study participants (WM phase). At the end of the 12-week WL, participants selected themselves to either a modified (see below) P-CR (mP-CR) dietary program or HH diet for the remaining 52 weeks.

## 3. Dietary Intervention

### 3.1. Weight Loss (WL) Phase (Weeks 1–12): P-CR Diet

Beginning at week 1, subjects consumed a P-CR diet six days per week along with an intermittent-fast (IF) day on the remaining day. The dietary products and daily energy intake on P-CR consisted of the following: A 240 kcal liquid meal replacement shake (containing 24 g dairy protein) for both breakfast and lunch, a 150 kcal afternoon snack (men only), and a 450 kcal or 600 kcal whole-food dinner, for women and men, respectively. Finally, a 250 kcal meal replacement bar (containing 18 g dairy protein) was provided as an evening snack. This program provided approximately 1200 and 1500 kcals per day for women and men, respectively, along with a distribution of 30% protein, 45% carbohydrate, and 25% fat (Table S1). This macronutrient distribution has previously been successfully utilized in our lab to induce an energy deficit without compromising lean body mass [8]. On the IF day (self-chosen), caloric intake consisted of six servings of an antioxidant-rich beverage providing 120 kcals, three servings of low-glycemic protein wafers providing 90 kcal, and a 100 or 200 kcal whole-food, high-protein snack for women and men, respectively (Table S2). Total caloric intake on IF days was approximately 350–450 kcals for women and men, respectively. All subjects also consumed a daily micronutrient supplement pack containing a combination of minerals and vitamins, phytonutrients/antioxidants, and essential fatty acids along with a nutrient-rich herbal tonic. All meal replacement shakes, bars, beverages, and dietary supplements were provided by Isagenix International, LLC (Gilbert, AZ, USA).

### 3.2. Weight Maintenance (WM) Phase (Weeks 13–64): Modified P-CR (mP-CR) or Heart Healthy (HH) Dietary Interventions

Following the 12 week WL phase, participants either self-selected to continue a modified P-CR dietary regimen or transition to a HH dietary intervention. For the mP-CR intervention, subjects followed a diet similar to the WL phase, but consumed food ad libitum, and two meals per day were meal replacements (either as two shakes, or as one shake and one meal replacement bar) while the remaining 2–3 meals were whole foods. mP-CR subjects were also required to continue the IF protocol but only 1–2 times per month. This mP-CR dietary approach was to provide a “real world” long-term maintenance approach to adherence to the program. The HH group observed the dietary guidelines that are in compliance with the National Cholesterol Education Program Therapeutic Lifestyle Changes (TLC) diet (i.e., <35% of kcal as fat; 50%–60% of kcal as carbohydrates; <200 mg/dL of dietary cholesterol; and 20–30 g/day of fiber). The HH subjects were provided a monthly food stipend to assist in complying with the TLC diet plan. All subjects (HH and mP-CR) had monthly meetings with a registered dietitian to help make healthy eating choices that were compliant with their meal plans. Additional counseling with the registered dietitian was made available to participants if necessary. In order to resemble a “free-living” pattern of energy balance, subjects were instructed to follow the guidelines of their respective diets without restriction on physical activity or total food intake. 

### 3.3. Compliance

To encourage compliance, all subjects had weekly meetings with a registered dietitian during WL and monthly meetings during WM to incorporate healthy eating strategies while adhering to the specifications of their dietary regimens. Additionally, all subjects were given detailed written/verbal instructions for each diet plan. Monitoring of the meal plans was performed through daily subject-researcher interaction (e.g., telephone conversations), two-day food diary analysis, weekly dietary intake journal inspections, distribution of weekly meal/supplement containers and return of empty packets and containers. The researchers held weekly meetings with all participants to verify compliance with the dietary meal plans, clarify dietary guidelines, and answer questions. Participants demonstrated a high compliance rate (>90%), which was defined as consuming more than 85% of their respective meals/supplemented feedings. Subjects were considered noncompliant if they were absent from more than two consecutive dietitian meetings or consuming ≥3 inappropriate meal/supplement servings a week for ≥2 consecutive weeks at a time.

A two-day food record was utilized to verify compliance to each diet. Food records were filled out by the participants on weeks 0, 11, and 63. A registered dietitian and a research team member gave the participants instructions on making detailed records of portion sizes and food items. The dietary information was subsequently recorded into the food analysis program, The Food Processor SQL Edition (version 10.2.0 ESHA Research, Salem, OR, USA, 2012). A single trained operator performed the analysis in order to lessen inter-investigator variation. Each participant was also given a checklist in an effort to help them adhere to the CR-IF dietary regimen. 

## 4. Laboratory Testing Procedures

### 4.1. Body Composition Assessments

At weeks 0, 12, and 64, all participants were tested between the hours of 6:00 a.m. and 9:00 a.m., after an overnight fast, and underwent body composition assessments (height, body weight, as well as regional and total body composition). Body weight was obtained using a standard digital scale and height obtained without shoes using a stadiometer. Waist circumferences were obtained in centimeters with a standard tape measure. Waist measurement was obtained at the area with the smallest circumference between the rib cage and the iliac crest. Body composition was assessed by dual energy X-ray absorptiometry (iDXA; Lunar iDXA; GE Healthcare, Madison, WI, USA; analyzed using encore software version 13.6). Total body fat (TBF), % body fat, lean body mass (LBM), visceral adipose tissue (VAT), and regional abdominal fat (ABF) were all analyzed from iDXA scans as previously described (6). Test-retest intraclass correlation (*r*) and coefficient of variation (CV) for body composition analysis using iDXA in our laboratory with *n* = 12 is: FFM and FM *r* = 0.99, CV = 0.64%, and *r* = 0.98, CV = 2.2%, respectively, and for regional abdominal body composition analysis was: % fat *r* = 0.99, CV = 2.4% were recorded for each participant. Standard body mass index (BMI) measurements were obtained by dividing the subject’s weight (kg) by the square of their height (m^2^).

### 4.2. Physical Activity Assessment

Baseline energy requirements were calculated for each individual by physical activity energy expenditure (Bio-Lynx Scientific Equipment Inc., Montreal, QC, Canada) and indirect calorimetry (ParvoMedics, TrueOne 2400, Parvo, UT, USA). In addition, subjects were asked to follow their routine eating patterns and record dietary food logs for two days during baseline control. Likewise, during the WL phase subjects were asked to maintain their current level of physical activity (sedentary/low activity) and to abstain from starting any new exercise programs. In order to verify sedentary/low activity levels, all participants wore an Actical accelerometer (Bio-Lynx Scientific Equipment Inc.) around their waist for two days during weeks 0, 10, and 62.

### 4.3. Plasma Biomarkers

For adiponectin, leptin, insulin and glucose measurements, 12-h fasted venous blood samples (~20 mL) were collected into EDTA-coated vacutainer tubes and centrifuged (Hettich Rotina 46R5) for 15 min at 2500 rpm at 4 °C. After separation, plasma was stored at −70 °C until analyzed. Plasma concentrations of insulin, leptin and adiponectin were analyzed by ELISA, and glucose determined using a glucose oxidase protocol (GM7 Analyser, Analox Instruments, Lunenberg, MA, USA). Test-retest intraclass correlation (*r*) and coefficient of variation (CV) in our laboratory with *n* = 15 was: Insulin, and glucose (mg/dL) *r* = 0.95, CV = 3.2%, and *r* = 0.97, CV = 5.3%, respectively.

### 4.4. Resting Metabolic Rate (RMR)

Subjects rested in a supine position for 15 min in a quiet and dimly lit room for a 30-min resting metabolic rate (RMR) measurement. Resting metabolic rate was measured via indirect calorimetry at weeks 0 and 13 using the ventilated hood technique (ParvoMedic; analyzed via True One 2400 software). Participants arrived at the Human Nutrition and Metabolism Laboratory immediately upon waking (between 0600 and 0800). Test-retest intraclass correlation (*r*) and coefficient of variation (CV) in *n* = 14 was: RMR (kcal/min) *r* = 0.92, 4.2%, respectively.

### 4.5. Dietary Intake and Feelings of Hunger and Satiety

Throughout the intervention, subjects maintained a daily food log that included all food and beverages consumed each day, including meal timing. To further verify compliance, food intake was analyzed from a representative 3-day period at weeks 0 and 12 during WL and week 26 during WM using Food Processor SQL Edition (version 10.12.0, 2012; ESHA Research, Salem, OR, USA) as previously described [21]. All dietary analyses were performed by the same technician. Visual analog scales (VAS) were administered at weeks 0, 12 and 64 to evaluate the effects of the lifestyle interventions on hunger, satiation, and desire-to-eat [22]. Briefly, participants were instructed, using a pen and paper, to mark their levels of hunger, satiety, and desire-to-eat on a 100 mm line that was anchored at either end with “0” (none) to “100” (extreme). For each of these three measures the degree of sensation was quantified by the distance of the mark from the “0” mm point. 

### 4.6. Statistical Analysis

Statistical analysis was performed using SPSS software (Version 21; IBM-SPSS, Armonk NY, USA). Before the start of the study, sample size was determined through power analysis based on the major outcome variables body weight, body composition, and energy expenditure to achieve an effect size of 0.25 with 80% power at alpha 0.05 based on previous data [8,14,21]. This analysis determined that *n* = 12 were required to detect a significant mean difference of 1.4 kg weight loss between the two diet intervention groups (mP-CR vs. HH) during WM. Absolute changes in body weight (kg) and composition, biomarkers, metabolism (RMR, RQ), and hunger ratings were calculated. A 2 × 2 factor repeated measures ANOVA was performed for the WL Phase 1 (P-CR, weeks 0–12) using sex (men vs. women) and time (week 0 vs. 12) and the WM Phase 2 (weeks 13–64) using group (mP-CR vs. HH) and time (week 13 vs. 64) to determine main effects. Where significant main effects were identified, post hoc comparisons (Tukey’s test) were performed to locate differences. Data analysis was not performed blinded but each intervention group was assigned with a number code. One-Tailed tests were utilized for this study and the significance was set at *p* < 0.05. All values are reported as means ± SE unless stated otherwise.

## 5. Results

### 5.1. Weight Loss Phase 1 (WL; P-CR, Weeks 0–12)

#### Subject Characteristics

Three individuals did not adhere to the P-CR dietary guidelines and were dropped due to non-compliance. Thus, descriptive baseline characteristics of the forty subjects (19 females and 21 males) who completed WL phase 1 are reported in Table 1. 

### 5.2. Dietary Intake during WL (Weeks 0–12)

The P-CR WL diet intervention significantly altered dietary energy and macronutrient intake for both sexes (Table 2). 

Total energy intake following P-CR diet decreased significantly (*p* < 0.05) in both females and males by ~50% during the WL phase, with no differences between the sexes. This reduction was due to considerable decreases in percentage of dietary fat (12%–15%, *p* < 0.05) and total carbohydrate intake (<175 grams/day; 50% drop in g/day, *p* < 0.05) intake. However, percentage of calories from protein intake increased significantly (17% ± 1% vs. 32% ± 1%, *p* < 0.05) in both men and women. Other macronutrients significantly affected by P-CR diet included increased dietary fiber (6 g/day) and reductions in sugar (>55 g/day) and sodium (~3000 mg/day) intake.

### 5.3. Body Weight and Composition during WL (Weeks 0–12)

The effect of the P-CR protocol on body weight and body composition is shown in Table 3.

Relative to baseline, both females and males exhibited similar statistically significant reductions (*p* < 0.05) in all measured parameters (body weight, waist circumference, total body fat, fat mass, lean body mass, and abdominal and visceral adipose tissue mass, VAT) after the WL phase. Average weight loss for women and men following P-CR diet was 10.3 and 12.7 kg or 10.4% and 10.6% weight loss from baseline, respectively. Similarly, over the 12-week P-CR diet intervention, subjects lost on average 9.4 kg (19%) total body fat, 1.3 kg (25%) abdominal fat, and 0.8 kg (33%) VAT. Although total LBM decreased during P-CR by ~1.7 kg; when expressed as a percentage of body weight, LBM increased by 8.7%, with no differences between men and women. 

### 5.4. Plasma Biomarkers during WL (Weeks 0–12)

Plasma biomarkers also responded significantly to the P-CR diet when compared to baseline values for both men and women. Fasting glucose dropped by 12% and 7% and insulin by 40% and 42% for men and women, respectively (*p* < 0.01), with no differences between the sexes (Table 4).

Leptin decreased 51% and 73% (*p* < 0.01) in men and women, respectively. In contrast, adiponectin levels were unaffected during the WL phase. There were no differences between sexes for any plasma biomarkers.

### 5.5. Resting Metabolic Rate (RMR), Physical Activity and Hunger Ratings during WL (Weeks 0–12)

RMR increased significantly in both sexes when expressed as kcals/kg body weight (BW) and respiratory quotient (RQ) decreased 9% (Table 5). There were no sex differences for any metabolic, physical activity, and hunger ratings in response to the P-CR diet during the WL phase. 

### 5.6. Weight Maintenance Phase 2 (WM; mP-CR vs. HH; Weeks 13–64)

#### Subject Characteristics

After subjects completed the WL phase of the P-CR dietary intervention, those that continued the study self-selected themselves into either a modified P-CR (mP-CR) or heart healthy (HH) dietary plan for phase 2, WM. Twenty five subjects completed the 52-week WM intervention. A total of 13 subjects dropped out during the intervention: nine of nineteen mP-CR participants (47%) vs. four of eighteen in the HH diet (22%). The reasons for the difference in compliance ranged from scheduling conflicts, non-compliance of participants and health reasons. Participants who completed phase 2 are highly motivated and devoted to the study, and hence, are more motivated to comply with the study. Unfortunately, data for those who were lost to follow-up is not available since it was not collected and, therefore, potentially reduced the possibility of false positives. Importantly, this did not affect the significance of the results since there is no significant difference in terms of the baseline measurements between the groups for WM. Because there were no significant sex-specific responses to the WL P-CR protocol (Phase 1), men and women were pooled together for WM (Phase 2). As a result, there were no differences in body weight, body composition, or any other parameter between groups at the time of treatment designation (week 13). 

### 5.7. Dietary Intake during WM (Weeks 13–64)

No differences existed between mP-CR and HH during the WM intervention with the exception of dietary sodium intake, which was greater in HH (Table S3).

### 5.8. Body Weight and Composition during WM (Weeks 13–64)

**Figure 3:**
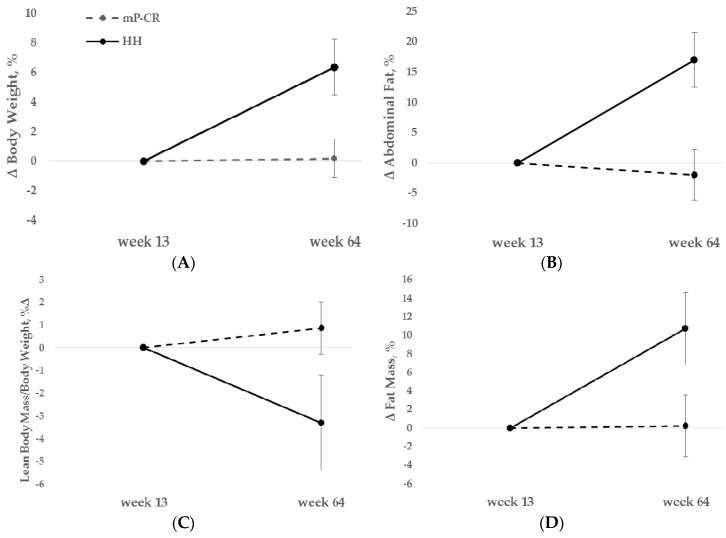
Body composition changes between modified protein-pacing, caloric-restriction (mP-CR) and heart healthy (HH) dietary groups during the WM period (weeks 13–64) expressed as body weight (**A**); abdominal fat (**B**); lean body mass/body weight (**C**); and total fat mass (**D**) changes. Mean ± SE.

Long-term adherence to mP-CR and HH dietary groups during WM resulted in significant differences in body weight and body composition parameters (Table 6; Figure 3A–D).

Specifically, mP-CR participants gained significantly less weight compared to the HH group (0.6% ± 1.9% vs. 6.1% ± 2.1%, *p* < 0.05) (Figure 3A). The change in % body fat was also significantly lower (−0.4% ± 2.0% vs. 5.8% ± 2.0%, *p* < 0.05) in the mP-CR group than the HH group. Changes in abdominal fat, lean body mass, and fat mass were significantly reduced in mP-CR compared to HH following (Week 64) the WM intervention (Figure 3B–D).

### 5.9. Individual Responses in Body Weight and Fat Mass

**Figure 4:**
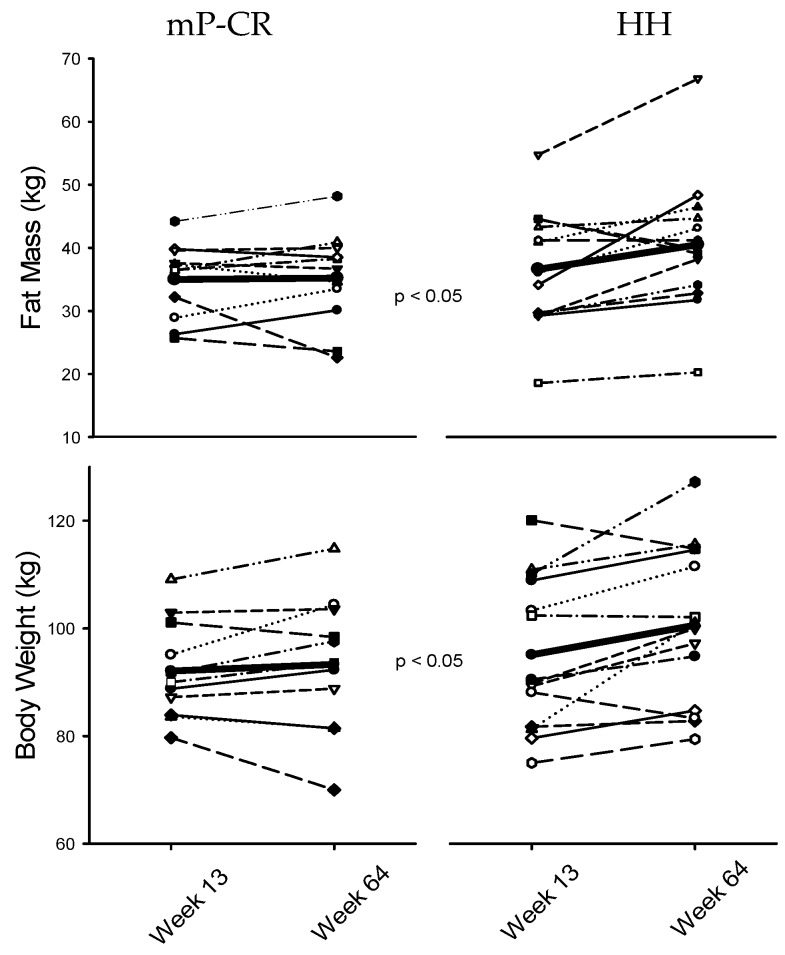
Individual changes in fat mass and body weight during WM (Phase 2) between mP-CR and HH.

Waist circumference also tended to increase to a lesser extent in mP-CR compared to HH (mP-CR, 98.2 vs. 101.4; HH, 97.6 vs. 104.6 cm, *p* = 0.075) (Figure 4).

### 5.10. Plasma Biomarkers during WM (Weeks 13–64)

Plasma glucose and insulin levels did not change during the 12 month WM phase for either mP-CR or HH dietary groups (Table 7). Due to logistics, leptin and adiponectin were obtained half-way through the WM intervention (39 weeks) and not at post-intervention (week 64). Interestingly, both leptin and adiponectin increased significantly over the course of the first 6 months of WM in both mP-CR and HH groups.

### 5.11. Resting Metabolic Rate, Physical Activity and Hunger Ratings during WM (Weeks 13–64)

RMR expressed as an absolute change and kcals/BW was unaffected by either dietary protocol during the WM phase, however, respiratory quotient increased significantly in both groups (Table 8). Additionally, both “hunger” and “desire to eat” increased over the WM intervention in both groups (*p* < 0.05).

## 6. Discussion

The two primary aims of this study were: (1) to examine sex-specific responses to short-term (weeks 0–12) WL, including body composition, resting metabolic rate (RMR), and biomarkers following a P-CR diet intervention in obese men and women and (2) to extend beyond the WL phase and include a long-term (weeks 13–64) WM phase to compare a mP-CR to a HH dietary intervention. The main findings of the current study demonstrate that obese men and women respond similarly to a 12 week P-CR WL diet in terms of: (1) significant reductions (*p* < 0.01) in weight (10%), TBF (19%), ABF (25%) and VAT (33%), glucose (7%–12%), insulin (42%), leptin (>50%) and increase in proportion of LBM (9%); (2) RMR (kcals/kg body weight) increased 5% and RQ decreased 9%; and during long-term (52 weeks) WM; (3) the mP-CR diet results in significantly (*p* < 0.05) lower body weight (6%), TBF (10%), and ABF (17%) as well as greater LBM (5%) compared to a HH dietary intervention. 

Taken together, our results suggest that a short-term (12 weeks) P-CR weight loss diet intervention is equally effective at enhancing all aspects of body composition (total, abdominal and visceral fat mass; lean body mass), plasma biomarkers (insulin, glucose, leptin) and metabolism (RMR, RQ) in obese men and women. In addition, following a long-term (52 weeks) WM intervention, mP-CR is more efficacious at preventing recidivism of body weight and composition (fat mass, abdominal fat, lean body mass) compared to a traditional HH diet intervention.

### 6.1. Weight Loss, Phase 1 (WL; P-CR, Weeks 0–12)

#### 6.1.1. Body Weight and Composition

A primary aim of this study was to investigate sex specific responses to WL, specifically total and abdominal (including visceral) fat mass in obese men and women following a short-term P-CR intervention. Herein, we report a P-CR diet effectively reduced body weight, total and abdominal (including visceral) fat mass in both men and women with no significant sex difference. Both sexes were included in the current study since our previous work has suggested men and women, in similar fashion, significantly reduce total and abdominal (visceral) fat mass in response to a high protein low calorie/carbohydrate diet [8]. Unfortunately, much of the previous P-CR research has been skewed toward women rather than men [14,16]. Short-term P-CR interventions have previously demonstrated to effectively induce weight loss and improve overall body composition [14]. However, men suffer from a higher prevalence of cardiovascular complications compared to women, indicating that sex-based differences may play a key role in fat metabolism and cardiometabolic health [23,24]. Other studies suggest that men lose more VAT than women during weight loss [19,20], although this is not universal [8,20]. Interestingly, there were no significant differences between males and females in body weight, waist circumference, TBF and ABF (including VAT), and LBM after the WL phase.

Indeed, following the WL phase, the proportion of LBM actually increased by 9%, whereas TBF (19%), ABF (25%), and VAT (33%) decreased significantly (*p* < 0.01). In comparison to previous caloric restriction diet interventions, this finding is very promising. Current evidence suggests that the energy deficit likely reduces basal muscle protein synthesis (MPS) [25] and may also have reduced the sensitivity of MPS to feeding [26,27]. Nonetheless, recent data have shown that lower rates of MPS can also be restored by a higher dietary protein intake [28], particularly so with whey protein [26], which was the supplemental protein source used throughout the WL phase. In support of the current findings, we have previously shown the benefits of P-CR in overweight individuals and reported significant improvements in TBF, ABF, and LBM following a 4-week P-CR diet (35% PRO over 6 meals/day) when compared against a traditional HH dietary intake of protein (15% PRO over 3 meals/day) [8]. It is likely that the reduced absolute level of carbohydrate (<175 grams/day) also played a significant role.

#### 6.1.2. Plasma Biomarkers

Following the WL phase (P-CR, weeks 0–12), glucose, insulin and leptin significantly decreased while adiponectin was unaffected. Leptin and insulin are often referred to as “adiposity signals” because their levels generally reflect fat mass. Fasting levels of both hormones decreased with the decline in adiposity that occurred with weight loss (Table 4). The significant reduction in dietary carbohydrate intake (50% reduction; <175 grams/day) and caloric restriction are well documented factors for the improved glucose and insulin response following P-CR induced WL. Caloric restriction has also been shown to reduce circulating levels of leptin [29,30] and the decline in leptin is more intuitive because it is secreted directly from adipocytes while the impact on insulin is indirect, reflecting the improvement in insulin sensitivity that occurs with weight loss [31]. In a 10-week CR trial in obese women, Kroeger et al. [16] found a 25% decrease in leptin, which were similar to findings from Harvie et al. [32]. The greater reductions in leptin in the current study (51% men, 70% women) are most likely due to the greater fat loss achieved compared to the previous studies. Interestingly, circulating leptin levels are also directly proportional to the total amount of visceral fat in the body [33], which corroborates the improvement observed in the current study. 

Adiponectin is a fat-cell-derived hormone shown to be inversely related to body weight and visceral fat mass, though the data are not consistent. For example, Klempel and Varady [34] noted an association with adiponectin with a greater than 10% weight loss, however results were variable [34]. Interestingly, in the present study, a 10% weight loss was achieved in both men and women and adiponectin did not change significantly. A previous investigation indicated visceral fat mass is negatively correlated with circulating adiponectin but this relationship is greatly diminished in women over 40 years old [35]. Thus, the plausible explanations for no change in adiponectin in the current study was weight loss did not exceed 10% and the older age of our subjects, both of which may have influenced the responsiveness of adiponectin to the dietary intervention, at least over this 12-week period. Clearly, more research needs to be conducted regarding the threshold necessary to induce a meaningful change in adiponectin following a P-CR diet intervention in middle-aged obese adults.

#### 6.1.3. Resting Metabolic Rate, Physical Activity and Hunger Ratings

One of the more intriguing findings of the current study was a meaningful 5% increase in RMR on a relative basis (kcals/kg body weight) in both men and women following a ≥10% weight loss due to the P-CR intervention. A plausible explanation for the enhanced metabolism (kcals/kg body weight) may have been due to the protein-pacing [8], leading to an increased proportion of lean body mass (9%) triggered by enhanced skeletal muscle protein synthesis [28]. It is well known that lean body mass is the single greatest predictor of RMR and these two paralleled each other during the WL intervention. Additionally, the favorable changes in RMR and LBM occurred concomitant to a decrease in RQ (increased fat oxidation) in both the men and women. Clearly, the favorable metabolic effects of the protein-pacing warrants further investigation.

Of special note, despite a 50% reduction in total kcals consumed during the P-CR WL intervention, feelings of hunger and satiety were unchanged in both obese men and women. This finding has potential implications for future recommendations as an effective weight loss strategy for men and women.

### 6.2. Weight Maintenance, Phase 2 (WM; mP-CR vs. HH, Weeks 13–64)

#### 6.2.1. Body Weight and Composition

A unique approach of the current study was to investigate changes in body weight, TBF, ABF, and LBM by comparing a m-PCR to a traditional HH diet during a 52 week (weeks 13–64) WM follow-up after the initial 12 week WL phase (Phase 1, WL; P-CR, weeks 0–12). This component was separated as its own phase (Phase 2, WM; mP-CR vs. HH, weeks 13–64). Since no significant differences existed between men and women for any variable following Phase 1, participants were pooled together at the start of Phase 2 (week 13). A novel outcome of the WM Phase 2 was the significantly smaller weight regain at the end (week 64) in participants following the mP-CR protocol compared to those adhering to the traditional HH diet. Specifically, the data shows <1% increase in body weight in the mP-CR group versus a ten-fold increase of 6.1% in the HH group at the end of WM (week 64). Perhaps more impressive was the continued maintenance of TBF and ABF loss and proportion of LBM in mP-CR versus the body weight and composition recidivism in the HH group (Figure 3A–D). The observed recidivism in HH is a common consequence following diet-induced weight loss interventions, especially longer-term (1–2 years) traditional follow-up interventions using ad libitum designs that also include an initial rapid weight loss [36,37]. 

Although WL is readily achieved through CR in the short-term, few obese people maintain the WL long-term [38], due to lack of adherence [39,40]. In the present study, the ability of obese subjects to maintain meaningful WL (≥10% of total body weight lost) and enhanced body composition long-term (52 weeks) was compared using two different dietary interventions (mP-CR vs. HH). Interestingly, many of the favorable changes observed following short-term WL (Phase 1, P-CR) persisted for 52weeks only in the mP-CR and not the HH participants, suggesting that the quality of the diet is an important feature of WM in previously obese subjects. It is important to highlight that no differences existed in any dietary variable (with the exception of sodium intake) between the mP-CR and HH diet (Table S3). The only major differences between the diet interventions were the mP-CR, including the 1–2 days/month of IF. Thus, the novelty of the findings from this study support the quality micro/macro-nutrients provided in the diet to augment long-term WL maintenance and enhanced body composition in previously obese subjects.

#### 6.2.2. Plasma Biomarkers

During the WM phase (mP-CR vs. HH, weeks 13–64), insulin and glucose were unchanged, suggesting the enhanced insulin sensitivity that occurred following acute WL (weeks 0–12) was preserved throughout WM. This was somewhat surprising given the significant regain of body weight and TBF in HH participants and implies some other mechanisms are responsible for mediating insulin and glucose levels. Due to logistical factors, leptin and adiponectin were only measured at week 39 and both increased from week 12. The rebound in leptin is not surprising as this reflects the energy storage (fat) depot status in the body [33], whereas, the increase in adiponectin warrants further study. 

#### 6.2.3. Resting Metabolic Rate, Physical Activity and Hunger Ratings

Regaining weight after CR has also been related to adaptive thermogenesis, reduced energy expenditure, and changes in appetite mediating hormones [41,42]. In lean and obese adults studied during or shortly (<3 months) after WL, significant reductions in energy expenditure beyond those predicted solely on the basis of changes in weight and body composition have been documented [41]. After WL, changes in circulating levels of several peripheral hormones involved in the homeostatic regulation of body weight may be involved. In a 10-week WL very-low-calorie-diet (VLCD) program, circulating levels of leptin, ghrelin, glucagon-like polypeptide-1 (GLP-1), cholecystokinin (CCK), peptide tyrosine tyrosine (PYY), gastric inhibitory polypeptide (GIP), amylin, pancreatic polypeptide, and insulin, as well as subjective ratings of appetite were examined [42]. Subjects who achieved 10% WL after 10 weeks were then put on a one year maintenance diet where a greater-than-predicted decline in 24-h energy expenditure persisted even after some weight was regained. Therefore, the results of our study are interesting due to the vast differences in weight regain between groups but a similar RMR kg/body weight in the WM phase. This warrants further study.

Our finding that mP-CR significantly attenuated increases in body weight and TBF and ABF compared to HH despite similar maintenance in RMR following WM, may imply that P-CR has an additional ‘metabolic advantage’ in preventing weight relapse. Indeed, previous research from our laboratory confirms the metabolic and body composition advantages of protein-pacing in obese subjects, possibly due to the increased amount and timing of protein intake in the P-CR diet compared to traditional HH (lower protein component). Several recent studies lend support of the “high-protein” theory for effective WL and WM by demonstrating its long-term efficacy when incorporated in the diet [43] and the lack of preventing weight regain when it is not included [37]. The current study clearly supports mP-CR as being more effective at preventing fat mass and body weight relapse during WM. Thus, there exists a macronutrient-specific effect on total weight and fat mass loss at the end of a 1-year ad libitum P-CR follow-up intervention. It is worth noting that the “modified” P-CR used during WM in the current study incorporated an IF for only 1–2 days per month (which still provided 350 and 450 kcals/day for women and men, respectively). The IF was not intended for continued weight loss during the WM phase but was included to more closely mimic the P-CR diet that was used during the WL phase without the intended weight loss. Moreover, both P and CR induce significant health benefits and therefore was a logical diet intervention to compare to the HH control diet.

Several P-CR weight-loss intervention studies and reviews have consistently reported a higher protein component of solid food or drink induces greater satiety and delay appetite sensations at later meals than other macronutrients [9,44,45,46]. Although no differences were noted in hunger ratings during the current WM phase, in a recent meta-analysis, higher protein preloads were found to increase fullness ratings more than lower protein preloads under tightly controlled conditions [47]. This macronutrient-specific effect is mediated by gut hormones regulating satiety, such as GLP-1, CCK, and PYY [9,44,45,46]. It is likely the 1 or 2 days/month of IF provided an additive role in facilitating the WL maintenance mP-CR, which may be an interesting area for future studies. Another alternative explanation may be that the amount of rapid WL (approximately 10 kg) following Phase 1 may be difficult to maintain using a HH diet that does not incorporate P-CR and IF.

### 6.3. Strengths and Limitations

Major strengths of the present study include: (a) two separate phases (WL; WM) with two separate baselines and post-testing (weeks 0–12; weeks 13–64) for direct comparison within and between interventions; (b) carefully monitoring the diet and physical activity level of all subjects throughout the 12 week WL and 52 week WM phases; and (c) standardization of all measurements and laboratory procedures. There are several limitations associated with the current study. Abnormal metabolic status associated with obesity was likely impacted by our WL interventions. Indeed, our findings would be strengthened by a more comprehensive inclusion of metabolic status assessment and evolution (e.g., triglyceride level, blood pressure, blood glucose levels, cholesterol levels, resting metabolic rate, etc.) during WL and WM phases that compares mP-CR to HH. This should be an area of future research. Nine of nineteen mP-CR participants (47%) dropped out of the WM phase, versus four of eighteen in the HH diet (22%), the reason of which warrants further investigation. Due to the nature of long-term human studies, the total number of participants in Phase 2 is less than Phase 1. The reasons include drop-out, scheduling conflicts, non-compliance of participants, etc. Participants who completed Phase 2 are highly motivated and devoted to the study. Indeed, less motivated volunteers are likely to drop out. Unfortunately, data for those who were lost to follow-up is not available since it was not collected, therefore, potentially reducing the possibility of false positive. However, it does not affect the significance of the results since there is no significant difference in terms of the baseline measurement. Finally, the participants in this study benefited from weekly contact, which helped ensure compliance, but may also increase the risk of investigator bias. People adopting this diet regimen outside of a research study will not have such daily monitoring and so our results may not predict the weight loss and maintenance of standard users of this diet. In this case, further studies should evaluate the relative efficacy of free-living obese adults following a P-CR over longer intervention periods (>1 year) and compared to other dietary practices.

## 7. Conclusions

In summary, our results provide compelling support that a short-term (12 weeks) P-CR weight loss diet intervention is equally effective at enhancing all aspects of body composition (total, abdominal and visceral fat mass; lean body mass), plasma biomarkers (insulin, glucose, leptin) and metabolism (RMR, RQ) in obese men and women. Further, following a long-term (52 weeks) weight maintenance intervention a mP-CR is more efficacious at improving body weight and composition (fat mass, abdominal fat, lean body mass) compared to a traditional HH diet intervention and thus should be considered as a viable public health recommendation to facilitate weight loss and prevent weight recidivism in obese men and women.

## Figures and Tables

**Table 1:** Baseline (week 0) characteristics of participants for WL (Phase 1).

Variable	Men (*n* = 21)	Women (*n* = 19)	Total (*n* = 40)
Age (years)	46 ± 1.5	50 ± 2.3	48 ± 9
Height (cm)	178.9 ± 1.7	163.0 ± 1.0	171.4 ± 1.6
Weight (kg)	120.1 ± 4.8	99.5 ± 2.8	110.3 ± 3.3
Body Mass Index (kg/m^2^)	37.5 ± 1.5	37.5 ± 1.1	37.5 ± 0.95
Body fat (%)	40.3 ± 1.4	51.0 ± 1.0	45.4 ± 1.2
Systolic blood pressure (mmHg)	128 ± 2	122 ± 3	125 ± 2
Diastolic blood pressure (mmHg)	82 ± 2	77 ± 3	79 ± 2
Resting heart rate (bpm)	65 ± 2	65 ± 3	65 ± 2

Values are means ± standard error (SE).

**Table 2:** Changes in dietary intake during WL (Phase 1).

Variable		Men (*n* = 21)	Women (*n* = 19)	Total (*n* = 40)
Energy (kcal)	Week 0	3085 ± 225	2304 ± 122	2714 ± 145
	Week 12 ^a^	1493 ± 11	1199 ± 7	1350 ± 25
Protein (%)	Week 0	17 ± 1	17 ± 1	17 ± 1
	Week 12 ^a^	32 ± 1	32 ± 1	32 ± 1
Protein (g)	Week 0	131 ± 11	95 ± 7	114 ± 7
	Week 12	123 ± 2	98 ± 2	111 ± 2
Fat (%)	Week 0	38 ± 2	35 ± 2	36 ± 1
	Week 12 ^a^	23 ± 1	23 ± 1	23 ± 1
Fat (g)	Week 0	136 ± 14	93 ± 9	116 ± 9
	Week 12 ^a^	38 ± 2	31 ± 1	35 ± 1
Carbohydrate (%)	Week 0	43 ± 2	48 ± 2	45 ± 1
	Week 12	45 ± 1	44 ± 1	45 ± 1
Carbohydrates (g)	Week 0	341 ± 28	273 ± 14	309 ± 17
	Week 12 ^a^	170 ± 3	135 ± 3	152 ± 4
Sodium (mg)	Week 0	4689 ± 484	3790 ± 415	4262 ± 326
	Week 12 ^a^	1089 ± 165	882 ± 142	988 ± 109
Fiber (g)	Week 0	27 ± 3	20 ± 1	23 ± 2
	Week 12 ^a^	32 ± 1	26 ± 1	29 ± 1
Sugars (g)	Week 0	126 ± 20	100.8 ± 47.7	114 ± 12
	Week 12 ^a^	66 ± 3	54.4 ± 9.8	60 ± 2

Values are means ± standard error (SE); ^a^ Significant time effect (week 0 vs. week 12), *p* < 0.05.

**Table 3:** Changes in body weight and composition during WL (Phase 1).

Variable		Men (*n* = 21)	Women (*n* = 19)	Total (*n* = 40)
Body weight (kg)	Week 0	120.1 ± 4.8	99.5 ± 2.8	110.3 ± 3.2
	Week 12 ^a^	107.4 ± 4.2	89.2 ± 2.6	98.7 ± 2.9
Waist Circumference (cm)	Week 0	123.8 ± 3.3	107.5 ± 1.7	116.3 ± 2.3
	Week 12 ^a^	106.9 ± 3.0	93.9 ± 1.7	100.9 ± 2.1
Total Body Fat (%)	Week 0	40.3 ± 1.4	51.0 ± 0.9	45.4 ± 1.2
	Week 12 ^a^	35.2 ± 1.5	46.9 ± 1.0	40.7 ± 1.3
Fat Mass (kg)	Week 0	47.5 ± 3.2	49.5 ± 2.2	48.4 ± 2.0
	Week 12 ^a^	37.5 ± 2.9	40.9 ± 2.1	39.0 ± 1.8
Lean Body Mass (kg)	Week 0	68.3 ± 1.7	46.9 ± 0.8	58.1 ± 2.1
	Week 12 ^a^	66.4 ± 1.6	45.5 ± 0.8	56.4 ± 2.0
LBM/BW (%)	Week 0	57.6 ± 1.6	47.4 ± 0.9	52.7 ± 1.1
	Week 12 ^a^	62.7 ± 1.6	51.4 ± 1.1	57.3 ± 1.3
Abdominal Fat (kg)	Week 0	5.7 ± 0.5	4.8 ± 0.2	5.3 ± 0.3
	Week 12 ^a^	4.2 ± 0.4	3.8 ± 0.2	4.0 ± 0.2
Visceral Adipose Tissue (kg)	Week 0	3.2 ± 0.3	1.5 ± 0.1	2.4 ± 0.2
	Week 12 ^a^	2.1 ± 0.2	1.1 ± 0.1	1.6 ± 0.1

Values are means ± SE; ^a^ Significant time effect (week 0 vs. week 12), *p* < 0.05.

**Table 4:** Changes in plasma biomarkers during WL (Phase 1).

Variable		Men (*n* = 21)	Women (*n* = 19)	Total (*n* = 40)
Glucose (mg/dL)	Week 0	103.3 ± 5.0	96.4 ± 3.3	99.8 ± 3.0
	Week 12 ^a^	91.3 ± 2.1	89.8 ± 2.3	90.5 ± 1.5
Insulin (μU/mL)	Week 0	9.2 ± 1.1	6.0 ± 0.5	7.6 ± 0.7
	Week 12 ^a^	5.5 ± 0.8	3.5 ± 0.3	4.5 ± 0.5
Leptin (ng/mL)	Week 0	13.6 ± 1.7	84.7 ± 17.7	45.4 ± 9.8
	Week 12 ^a^	6.6 ± 1.3	22.9 ± 3.5	13.9 ± 2.1
Adiponectin (ng/mL)	Week 0	16.1 ± 2.1	27.8 ± 5.3	21.7 ± 2.9
	Week 12	15.7 ± 1.6	27.3 ± 4.3	21.2 ± 2.3

Values are means ± SE; ^a^ Significant time effect (week 0 vs. week 12), *p* < 0.05.

**Table 5:** Changes in metabolism, physical activity and hunger ratings during WL (Phase 1).

Variable		Men (*n* = 21)	Women (*n* = 19)	Total (*n* = 40)
Resting Metabolic Rate (kcals/min)	Week 0	1.5 ± 0.3	1.1 ± 0.1	1.3 ± 0.3
	Week 12 ^a^	1.4 ± 0.2	1.0 ± 0.1	1.2 ± 0.2
Resting metabolic rate (kcals/kg BW)	Week 0	18.2 ± 0.5	16.0 ± 0.4	17.2 ± 0.4
	Week 12 ^a^	18.8 ± 0.4	17.2 ± 0.3	18.1 ± 0.3
Respiratory Quotient	Week 0	0.86 ± 0.01	0.87 ± 0.02	0.86 ± 0.01
	Week 12 ^a^	0.79 ± 0.01	0.78 ± 0.01	0.78 ± 0.01
Physical Activity (kcals/day)	Week 0	887.7 ± 98.9	771.9 ± 83.6	826.4 ± 54.0
	Week 12	867.8 ± 82.6	604.8 ± 51.1	728.5 ± 52.0
Hunger (mm)	Week 0	33.6 ± 3.9	31.0 ± 2.6	33.4 ± 2.4
	Week 12	33.6 ± 3.8	26.2 ± 3.4	30.1 ± 2.6
Desire to Eat (mm)	Week 0	35.4 ± 4.0	30.4 ± 4.3	33.0 ± 2.9
	Week 12	36.4 ± 4.0	24.3 ± 2.9	30.6 ± 2.7
Quantity to Eat (mm)	Week 0	50.5 ± 4.1	42.9 ± 3.3	46.9 ± 2.7
	Week 12	44.3 ± 3.5	35.6 ± 3.2	40.2 ± 2.5
Fullness (mm)	Week 0	32.3 ± 3.6	34.4 ± 4.6	33.3 ± 2.9
	Week 12	34.8 ± 4.0	37.0 ± 4.9	35.9 ± 3.1

Values are means ± SE; ^a^ Significant time effect (week 0 vs. week 12), *p* < 0.05.

**Table 6:** Changes in body weight and composition during WM (Phase 2).

Variable		mP-CR (*n* = 10)	HH (*n* = 14)
Body weight (kg)	Week 13	92.1 ± 2.7	95.1 ± 3.7
	Week 64 ^a,b^	93.3 ± 3.8	100.6 ± 3.9
Total Body Fat (%)	Week 13	39.4 ± 2.4	39.9 ± 2.3
	Week 64 ^a,b^	38.6 ± 2.9	41.6 ± 2.3
Fat Mass (kg)	Week 13	35.0 ± 5.9	36.5 ± 9.5
	Week 64 ^a,b^	35.2 ± 7.5	40.4 ± 10.9
Lean Body Mass (kg)	Week 13	54.1 ± 3.5	54.9 ± 3.3
	Week 64 ^a^	55.0 ± 3.7	56.4 ± 3.3
LBM/BW (%)	Week 13	58.4 ± 2.5	58.1 ± 2.4
	Week 64 ^a,b^	58.8 ± 2.5	56.2 ± 2.3
Abdominal Fat (kg)	Week 13	3.6 ± 0.2	3.5 ± 0.3
	Week 64 ^a,b^	3.6 ± 0.3	4.0 ± 0.3
Visceral Adipose Tissue (kg)	Week 13	1.4 ± 0.2	1.4 ± 0.2
	Week 64 ^a^	1.6 ± 0.2	1.6 ± 0.2

Values are means ± SE; ^a^ Significant time effect (week 13 vs. week 64), *p* < 0.05; ^b^ Significant group effect (mP-CR vs. HH), *p* < 0.05.

**Table 7:** Changes in plasma biomarkers during WM (Phase 2).

Variable		mP-CR (*n* = 9)	HH (*n* = 14)
Glucose (mg/dL)	Week 13	90.6 ± 2.2	89.9 ± 2.6
	Week 64	96.8 ± 3.9	90.7 ± 3.0
Insulin (μU/mL)	Week 13	4.6 ± 0.6	3.7 ± 0.4
	Week 64	4.6 ± 0.8	4.2 ± 0.5
Leptin (ng/mL)	Week 13	16.5 ± 4.8	8.9 ± 2.1
	Week 39 ^a^	18.3 ± 5.1	15.5 ± 2.7
Adiponectin (ng/mL)	Week 13	20.4 ± 4.0	19.0 ± 2.0
	Week 39 ^a^	35.5 ± 7.9	28.3 ± 3.0

Values are means ± SE; ^a^ Significant time effect (week 13 vs. week 39), *p* < 0.05.

**Table 8:** Changes in energy metabolism and physical activity during WM (Phase 2).

Variable		mP-CR (*n* = 11)	HH (*n* = 14)
Resting metabolic rate (kcals/min)	Week 13	1.2 ± 0.2	1.2 ± 0.2
	Week 64	1.2 ± 0.2	1.2 ± 0.2
Resting metabolic rate (kcals/kg BW)	Week 13	18.2 ± 1.9	18.4 ± 1.5
	Week 64	18.2±1.6	17.9 ± 2.2
Respiratory Quotient	Week 13	0.76 ± 0.01	0.79 ± 0.01
	Week 64 ^a^	0.82 ± 0.01	0.87 ± 0.01
Physical Activity (kcals/day)	Week 13	792 ± 118	670 ± 73
	Week 64	927 ± 176	913 ± 178
Hunger (mm)	Week 13	26 ± 3	28 ± 4
	Week 64 ^a^	32 ± 6	40 ± 5
Desire to Eat (mm)	Week 13	24 ± 4	30 ± 3
	Week 64 ^a^	31 ± 4	43 ± 7
Quantity to Eat (mm)	Week 13	38 ± 5	39 ± 3
	Week 64	37 ± 6	45 ± 6
Fullness (mm)	Week 13	41 ± 7	42 ± 5
	Week 64	33 ± 5	37 ± 6

Values are means ± SE; ^a^ Significant time effect (week 13 vs. 64), *p* < 0.05.

## Supplementary Materials

The following are available online at http://www.mdpi.com/2072-6643/8/8/476/s1, Table S1: Sample menus and meal timing for women and men during WL phase (Protein-pacing/caloric restriction, CR; Weeks 0–12). Menus were isocaloric for all women and men, respectively; Table S2. Composition of intermittent fasting day diet during WL Phase 1 ^a^; Table S3. Dietary intake, hunger, satiety and fullness ratings during WM (Phase 2).

## Abbreviations

The following abbreviations are used in this manuscript:

P-CR	protein-pacing, caloric restriction
mP-CR	modified protein-pacing, caloric restriction
HH	heart healthy
WL	weight loss
WM	weight maintenance
ABF	abdominal body fat
VAT	visceral adipose tissue
ADI	adiponectin
BMI	body mass index
iDXA	dual energy X-ray absorptiometry
LEP	leptin
RMR	resting metabolic rate
LBM	lean body mass
TBF	total body fat
%BF	percent body fat
