# Supplementary material for: Protein-Pacing Caloric-Restriction Enhances Body Composition Similarly in Obese Men and Women during Weight Loss and Sustains Efficacy during Long-Term Weight Maintenance
Source: Nutrients. 2016 Jul 30;8(8):476. doi: 10.3390/nu8080476 (PMC4997389; doi:10.3390/nu8080476)
Supplement: Supplementary file 1 [file nutrients-08-00476-s001.docx]

**Supplementary Materials: Protein-Pacing
Caloric-Restriction Enhances Body Composition Similarly in Obese Men and Women during Weight Loss and Sustains Efficacy during Long-Term
Weight Maintenance**

Paul J. Arciero, Rohan Edmonds, Feng He, Emery Ward, Eric Gumpricht, Alex Mohr,
Michael J. Ormsbee and Arne Astrup

**Table S1.** Sample menus and meal timing for women and men during WL phase (Protein-pacing/caloric-restriction, CR; Weeks 0–12). Menus were isocaloric for all women and men, respectively.

| **Variable** | **Women (1200 kcals/day)** | **Men (1500 kcals/day)** |
| --- | --- | --- |
| Breakfast  (0600–0800) | Liquid protein meal IsaLean^®^; 240 kcals, 24 g protein, 24 g carbohydrate, 5 g fat; Caffeine beverage e+^®^;  Multi-Vitamin/Mineral Ageless Essentials^®^; Anti-Oxidants Ionix^®^;  20 kcals | Liquid protein meal IsaLean^®^;  240 kcals, 24 g protein, 24 g carbohydrate, 5 g fat; Caffeine beverage e+^®^;  Multi-Vitamin/Mineral Ageless Essentials^®^; Anti-Oxidants Ionix^®^; 20 kcals |
| Lunch (1100–1300) | Liquid protein meal IsaLean^®^;  240 kcals, 24 g protein,  24 g carbohydrate, 5 g fat | Liquid protein meal IsaLean^®^; 240 kcals,  24 g protein, 24 g carbohydrate, 5 g fat |
| Mid-Afternoon snack (1400–1600) |  | Greek yogurt, fruit; 150 kcals, 20 g protein; 12 g carbohydrate; 4 g fat |
| Dinner (1700–1900) | Fish/Poultry/Beef, fresh vegetables, chopped nuts, dried fruit, olive oil, milk; 450 kcals, 25 g protein;  50 g carbohydrate; 17 g fat | Fish/Poultry/Beef, fresh vegetables, chopped nuts, dried fruit, olive oil, milk; 600 kcals, 25 g protein; 69 g carbohydrate; 25 g fat |
| Evening snack (2100–2200) | Protein bar IsaLean^®^; 250 kcals,  18 g protein; 27 g carbohydrate;  9 g fat | Protein bar IsaLean^®^; 250 kcals,  18 g protein; 27 g carbohydrate; 9 g fat |

**Table S2.** Composition of intermittent-fasting day diet during WL Phase 1 ^a^.

| **Whole-food high-protein snack** | **1/day** | **100 or 200 kcal for females and males, respectively** |
| --- | --- | --- |
| Anti-oxidant plant-based powder ^b^ | 6/day | 120 kcal total |
| Low-glycemic protein wafers ^c^ | 3/day | 90 kcal total |
| Micronutrient supplement ^d^ | 2/day | Contains vitamins, minerals, phytonutrients, antioxidants, and essential fatty acids |
| Plant-based herbal adaptogen powder ^e^ | 1/day | 20 kcal total |

^a^ During WL Phase 1, participants performed one day of IF per week; total energy intake of 330 kcal/day for women and 430 kcal/day for men. All supplements were provided by Isagenix LLC, Chandler, AZ, USA; ^b^ Cleanse for Life^®^; ^c^ Snacks™; ^d^ Ageless Essentials with Product B, AM & PM^®^, consumed on IF and non-IF days; ^e^ Ionix Supreme^®^, consumed on IF and non-IF days.

**Table S3.** Dietary intake, hunger, satiety and fullness ratings during WM (Phase 2).

| **Variable** | **mP-CR (*n* = 10)** | **HH (*n* = 14)** |
| --- | --- | --- |
| Energy (kcals/day) | 1788 ± 257 | 1624 ± 156 |
| Protein (%) | 28 ± 3 | 25 ± 2 |
| Protein (g) | 117 ± 5 | 101 ± 9 |
| Fat (%) | 29 ± 3 | 29 ± 3 |
| Fat (g) | 62 ± 16 | 56 ± 10 |
| Carbohydrates (%) | 42 ± 2 | 46 ± 3 |
| Carbohydrates (g) | 192 ± 31 | 182 ± 15 |
| Sodium (mg) * | 1639 ± 247 | 2838 ± 360 |
| Fiber (g) | 24 ± 3 | 22 ± 3 |
| Sugar (g) | 75 ± 13 | 65 ± 8 |

Note: PA, physical activity; P, protein-pacing; HH, heart-healthy. Data is presented as means ± SE. * Significantly different from Heart Healthy (*p* < 0.05).
